# Expectations and communication in opioid pain management: a qualitative study of patients' experience

**DOI:** 10.1080/02813432.2026.2616517

**Published:** 2026-01-23

**Authors:** Jennifer R. Amin, Elsa Ekelin, Emma Nilsing Strid, Katja Boersma, Sofia Bergbom

**Affiliations:** a Center for Health and Medical Psychology (CHAMP), Örebro University, Sweden and Research School for Integrated Care, Örebro University, Sweden; b University Health Care Research Center, Faculty of Medicine and Health, Örebro University, Sweden

**Keywords:** Chronic pain, communication, expectations, opioids, reflexive thematic analysis

## Abstract

**Background:**

Chronic pain remains a leading cause of patient distress in primary care and effective pain management presents an ongoing challenge in patient-clinician interactions. The prescribing of opioids further contributes to communication and shared decision-making disparities between patients and general practitioners. Gaining greater insight into patients' experiences of opioid treatment is valuable as there still is limited knowledge. Patients' perspectives and expectations can provide important contributions to enhance mutual understanding in clinical encounters.

**Aim:**

To explore how patients experience, and what they expect from, pain management consultations regarding opioid use.

**Design and setting:**

A qualitative study with patients in rural Örebro County, Sweden.

**Method:**

Semi-structured interviews were carried out with fifteen chronic pain patients prescribed opioids managed in primary care. The interviews were analyzed using reflexive thematic analysis.

**Result:**

Two main themes were generated to capture patients experience and their expectations concerning pain management and opioids. Prescribing Validation gives insights to what expectations patients have and how they perceive prescriptions. Renewals are interpreted as validation of the pain condition, and dismissals as mistrust. The Battle for the Steering Wheel capture how patients, based on the lived experience of chronic pain, expect and seek to assert their own expertise in consultations but often feel frustrated over being unheard.

**Conclusion:**

While education about the biopsychosocial nature of pain may provide a necessary foundation for communication around reducing opioid use, validation of patient experience is pivotal for building a trusting alliance.

Chronic pain is a complex illness causing significant suffering for individuals and presenting a challenge in patient–clinician interactions in primary care settings. Defined as pain persisting beyond three months, globally 10–30% of adults experience chronic pain [1–3]. The condition is one of the most common medical conditions in primary care [4–7] and is linked to increased mortality risks, comorbid health conditions [8,9] and associated with depression and anxiety [10,11]. The suffering of chronic pain can be long-lasting and one-fifth reported living with pain for over 20 years [1]. The condition has been linked to more frequent healthcare utilization, as shown by both systematic reviews of population data [12] and cohort studies capturing long-term usage patterns [13,14]. Further, general practitioners are identified as the health-care professionals responsible for the largest share of healthcare consultations about pain [12].

Optimal chronic pain management in primary care relies on a biopsychosocial and holistic approach [15]. Specifically, best practice highlights the value of GPs providing clear information and education, helping patients understand that persistent pain can occur without ongoing tissue injury or an active underlying disease process. This form of pain, termed nociplastic pain, is proposed to arise from altered processing at the level of the brain and spinal cord [16], where the pain system has become hyper-responsive, amplifying normal sensory input or generating pain without identifiable peripheral pathology [17,18]. Furthermore, GPs are thought to support self-management, encourage physical activity, address psychosocial factors and coordinate referrals to physical therapy and psychological therapies [15]. Due to risks and lack of effectiveness, guidelines do not stipulate opioids a preferred treatment for chronic pain but do state that opioids *can* be prescribed as a last resort for selected patients [19,20]. In sum, best practice guidelines for GPs management of chronic pain in primary care are highly complex and emphasize prioritizing nonpharmacological treatments, with pharmacological options used selectively and a restricted role for opioids due to the risk of adverse side effects.

However, opioids continue to be prescribed for chronic pain [21–23], a reflection of the complexity in managing long-term pain. National data from Norway, Sweden, and Denmark show that up to 12% of the population at least at one point received an opioid prescription [24]. Moreover, a clinical study with chronic pain patients (*N* = 703) set in UK primary care reported that 59% of participants had been prescribed one to eight opioid prescriptions over a 12-month period [25]. Nonetheless, when treating chronic pain, the goal is to improve functionality and quality of life, and for these purposes, there is not compelling evidence that opioids are effective [26–28].

Navigating the choice of opioids as a treatment option is a challenge for physicians. Conflicting perspectives between patients valuing opioids for pain relief and physicians concerns about adverse effects can result in conflict and communication disparities [29,30]. A recent meta-analysis concludes that symptoms indicative of opioid use disorder among patients prescribed opioids for chronic pain were estimated at 29.6%. This indicates that a proportion of patients may exhibit problematic opioid-related behaviors without meeting formal diagnostic criteria [31]. Addiction, sleep disturbances, constipation, nausea, and in severe cases, death have been reported as negative side effects of opioid consumption [32].

Although opioids have significant adverse side effects, patients tend to advocate for the benefits of these medications. Decreased pain or a way to control the pain, improve functioning, and reinforcement of independence are described as the benefits. However, to some degree, patients also acknowledge the various negative side effects [33,34]. Though opioids remain a widely prescribed treatment option they continue to be a subject of conflict between patients and healthcare providers creating a not fully understood tension in communication.

The complexity of communication regarding opioid treatment is described in Henry and Matthias [35] conceptual model of patient-clinician communication about pain (Figure 1). The core of the model is the interaction between patients and physicians where they exchange information and make decisions about treatment. The model proposes that patient-physician communication affects clinical outcomes and partially this is shaped by what the patients expect from their care and how they experience it. Despite the emphasis on putting the patient's experience in focus by adopting patient-centered care and implementing shared decision-making, a systematic review only found six studies focusing on chronic pain patients' perceptions of opioid use [36]. Thus, valuable insights could be gained by further exploring how patients experience communication and what they expect from opioid treatment in primary care.

**Figure 1.**
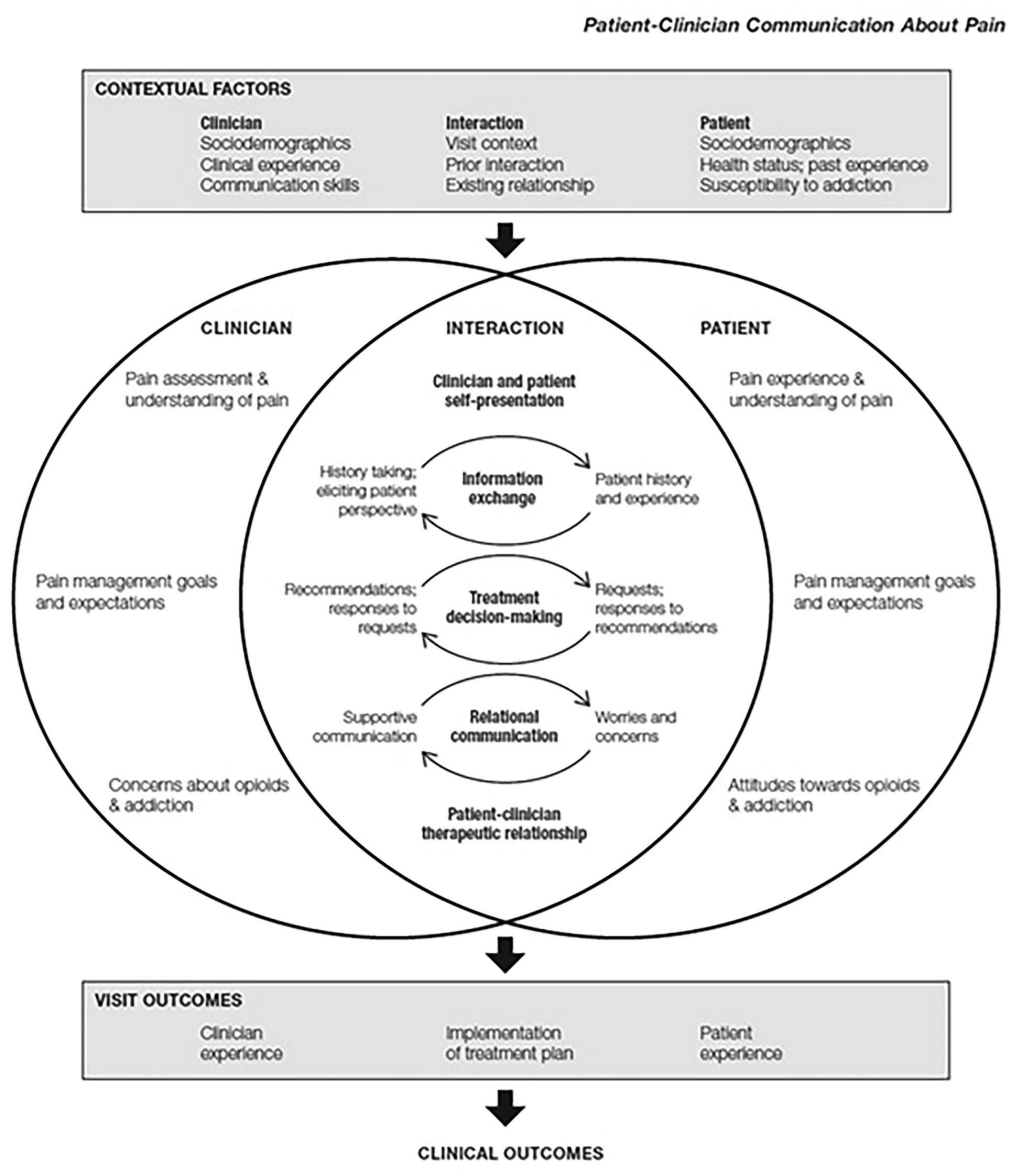
Conceptual model of patient–clinician communication about pain.

A part of communication is aligning patient expectations with the care offered. A broad range of studies indicate how positive expectations can influence pain and treatment outcomes [37–40]. This may be particularly important in the context of opioids, since patients often perceive them as an effective treatment [41]. Additionally, communication difficulties tend to escalate when opioid tapering is initiated, particularly when it is unexpected and not the patient's choice [42]. Researchers and healthcare providers are advised to recognize and explore different types of expectations that patients have to improve chronic pain healthcare outcomes [43]. Some expectations can be understood as *value expectations* [44] (e.g. wishes, hopes, what is seen as needed and what ideally should happen, including entitlements) and others as *predicted expectation*s [45] (e.g. expected results that the patient deem realistic). While some aspects of communication, like attentive listening by the physician, influence patients expectations [46], there still remains a gap in the research on what other aspects may influence these expectations.

Consequently, the purpose of this study is to contribute to the understanding of how patients experience their consultations with general practitioners (GP), with an emphasis on opioid treatment. The research questions posed were:

- How do patients experience their pain management consultations regarding opioid use?
- How do they describe the communication and their expectations regarding the consultation and opioid treatment?

## Method

### Design

This study was conducted in a primary care setting at mostly rurally located healthcare centers in Örebro County, Sweden and is part of a research project focused on understanding the patient-general practitioner relationship in the context of chronic pain management regarding opioid prescriptions. The overall study design, to be reported elsewhere, involved video-recordings of 20 patient-GP consultations, followed by video-stimulated recall interviews with the GPs and interviews with the patients. The consultations were held by nine GPs working at seven different care centers. Six GPs were male, the age range was 35–60 years (median = 49), five were educated in Sweden and four in Europe (except Sweden), working experience since license ranged between 6 and 36 years (median = 16), all were specialists in general medicine and one held a second specialty within rehabilitation and pain. The consultations lasted between 20 and 40 (mean 28) minutes.

This current study focuses on analyzing in-depth interviews conducted with patients after their consultations. The focus of the interviews was to explore the patients' experiences and perceptions of the interaction and communication with their GP regarding opioids.

### Participants and procedure

Recruitment and data collection were conducted between the 1^st^ of December 2020 and the 23^rd^ of November 2023. The research was approved by the Regional Ethics Board in Uppsala, Sweden (Dnr 2020–05561) and is in accordance with the ethical principles in the Declaration of Helsinki. The study applied purposive-convenience sampling and the inclusion criteria for patients were the following: (1) Aged 18–65, (2) registered at the healthcare center where the general practitioner works, (3) had been prescribed an opioid (defined by ATC codes N02AA, N02AB, N02AE, N02AG, N02AJ, N02AX, R05DA04) at some point during the past year, (4) able to speak Swedish with their general practitioner. Exclusion criteria for patients were active cancer treatment or palliative care. The recruitment process involved weekly monitoring of eligible patients for scheduled visits. 46 unique patients were identified, whereof 44 were reached. Participants who were reached received information regarding the purpose, aim and procedure of the study over the phone. They also received written information by email and were informed that they could withdraw consent at any given time without explanation. Out of the 44 who were contacted 20 interviewers were conducted. Reasons for rejecting participation were: (1) not wanting to be filmed, (2) high degree of psychosocial problems, (3) do not suffer from or do not want to discuss pain, (4) no reason. Participants were excluded from analysis if they no longer had opioid treatment, leaving eight women and seven men.

A semi-structured interview guide was developed by KB, ENS and EE (see Supplementary 2 for the interview guide), allowing participants to elaborate on their experiences regarding the consultation [47]. The topic areas were experiences, expectations, and goals of the consultation and with the pharmacological pain treatment; worries and concerns, specifically around opioids, addiction, decision-making, and relational communication.

All participants except one were interviewed over telephone by the second author, EE (GP and researcher in the overall study) after the consultation with their GP. The patients did not know that EE was familiar with or had worked with some of the GPs conducting the consultations. One interview was conducted by a psychology student involved in the project, also over the phone. The interviews were audio recorded and transcribed verbatim by professional secretaries and by the first author JA following the Jefferson Transcription System. JA also listened to the interviews multiple times and corrected some minor mistakes in the transcriptions. The interviews lasted 20 to 55 min. The interview transcripts were pseudonymized.

### Data analysis

This study applied a reflexive thematic analysis (RTA) approach [48,49] guided by a constructivist epistemological framework. This approach means viewing participants' narratives not as objective truths but seen as personal, shaped by their social context and healthcare experiences. The analytic process followed the six steps described by Braun and Clarke [49]. JA (first author) began the analysis by listening to all audio tapes and then reading all transcripts to familiarize herself with the data. Next, coding was completed by JA in the qualitative data analysis software NVivo 14. The coding involved both semantic and latent approaches with neither given precedence. Primarily an inductive approach was applied, meaning that the data was openly coded with a focus on the meanings derived from the participants and the data itself. However, some deductive analysis was also applied to ensure that the open coding helped generate themes that aligned with the research questions. This process ensured that the themes developed were both meaningful to the study's aim but also driven by the participant narrative during coding. Within RTA the concept of data saturation is not regarded as a fitting concept in relation to sample size. Saturation assumes a determinate set of discoverable themes, which is not aligned with the theoretically interpretative foundations of RTA. We had a narrow study aim and included participants with knowledge about the phenomenon, and the aim was that the dataset would be sufficiently rich and relevant to support a meaningful analytic narrative and not when no new information would” emerge”. The final sample provided the depth and scope needed to construct themes with shared meanings across the interviews [50].

All codes were compared, related codes were merged, and codes were classified into larger themes and subthemes. During this process of generating initial themes, reviewing themes and defining and naming the themes JA had ongoing discussions with co-author SB, an experienced pain researcher (PhD) and familiar with qualitative methods. SB was also engaged in reviewing themes compared to the data and defining and naming the themes.

This process was also developed through an ongoing, interpretive process, influenced by both participants' narratives and the researcher JA's theoretical orientation and reflexivity. The interpretative process involved constant reflexivity, knowing that the themes generated were shaped by the interaction between the data and the primary researcher JA's positionality. JA's is a female clinical psychologist from Sweden and a PhD student at a Swedish university. For more details and thoughts on how JA's background may have influenced the analysis, see the reflexivity statement. The results were also presented and discussed within the team consisting of the co-authors: 1) EE, a female PhD student and GP with experience from clinical work with pain patients, 2) ENS, a female associate professor and physiotherapist and 3) KB, a female professor in the field of pain research.

Excerpts are included in the results to enrich the narrative and ground the interpretations in participants' accounts.

## Results

Fifteen people were interviewed (eight women and seven men) and the average age was 53. The average duration of living with chronic pain was 22 years, all patients had an ongoing opioid prescription and the opioid discussion in the consultation regarded renewals. All but four were on sick leave or had disability pension. Further details of the participants' characteristics are seen in Table 1.

**Table 1.** Demographic data, characteristics and self-reported medical data, *(N = 15).*

mean (range) or n (%)	
Age (years)	53 (35–65)
Gender	
Women	8 (53)
Men	7 (47)
Education level^a^	
Elementary School or less	3 (21)
High school	8 (57)
University	3 (21)
Employment status*	
Full time	4 (27)
Sick leave	6 (40)
Disability pension	5 (33)
Pain conditions	
Lumbago (of which 5 disc disorder)	8 (53)
Fibromyalgia	2 (13)
Inflammatory bowel disease	1 (7)
Spinal stenosis	1 (7)
Myalgia	1 (7)
Multiple sclerosis	1 (7)
Reactive arthritis	1 (7)
Sarcoidosis	1 (7)
Juvenile osteochondrosis of spine	1 (7)
Cervicalgia after whiplash	2 (13)
Cervicalgia due to disc hernia	1 (7)
Shoulder/impingement	2 (13)
Migraine	1 (7)
More than one pain diagnoses	9 (60)
Duration of pain, years	22 (2–50)
>10 years	12 (80)
Opioids/Opiates^b^	
Codeine	4 (27)
Methadone	1 (7)
Oxycodone	8 (53)
Tramadol	3 [20]
Morphine	2 (13)
Opioid dose (MME)^c^	
≤ 10	8 (53)
11-50	4 (27)
> 50	3 (20)

^a^ *One of the participants did not have a formal degree of employment and could not estimate the working time*.

^b^ *Three patients were on a combination of opioids/opiates*.

^c^ *Opioid doses are reported in approximate morphine milligram equivalent (1, 2)*.

1. Walker PW, Palla S, Pei B-L, Kaur G, Zhang K, Hanohano J, et al. Switching from methadone to a different opioid: what is the equianalgesic dose ratio? Journal of palliative medicine. 2008;11(8):1103-8.

2. Nationellt kliniskt kunskapsstöd. Konverteringsguide opioider. 1177 Vårdguiden för vårdpersonal; 2023 Available from: https://vardpersonal.1177.se/globalassets/nkk/nationell/media/dokument/kunskapsstod/bilagor-kliniska-kunskapsstod/konverteringsguide-opioider-2023.pdf.

Two main themes were developed capturing how patients experience their pain management consultations regarding opioid use [1]: Prescribing Validation: The Role of Opioids in Shaping Perceptions of Care, and [2] The Battle for the Steering Wheel: The Patient's voice in Pain Management. Each theme reflects a different dimension of the patient's experience and illustrates how patients experience, interpret and react to their physicians' decisions concerning pain management with opioids.

**Figure 2.**
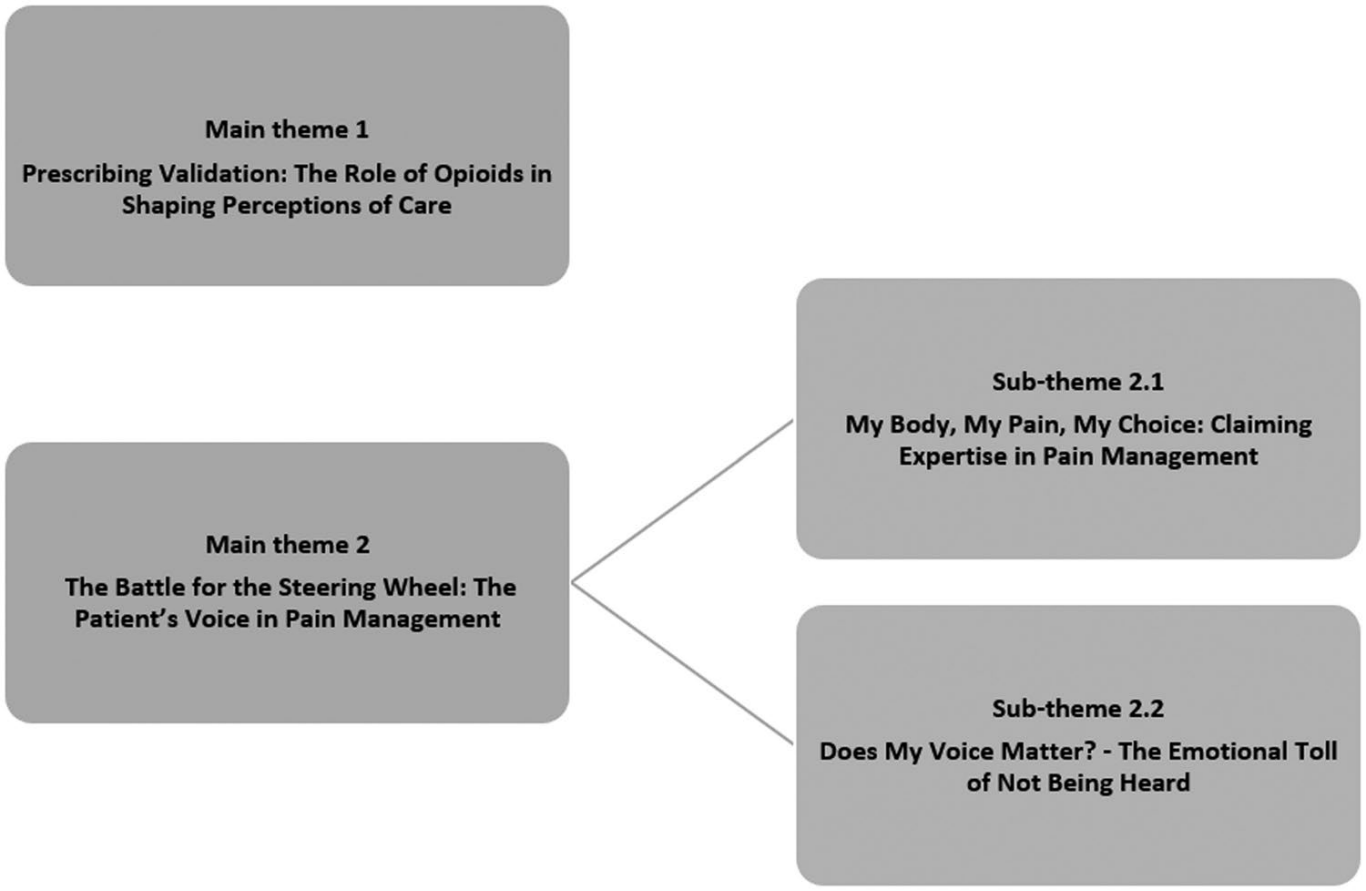
Themes and subthemes.

Main theme [1] Prescribing Validation encompasses how patients interpret prescription decisions. It captures the emotional experience of receiving or being denied opioids. In contrast, main theme [2] The Battle for the Steering Wheel focuses on how patients react in consultations, whether by asserting their own expertise or expressing frustration over being unheard. The themes and the subthemes are shown in Figure 2. Quotations are used throughout to exemplify themes in participants' own words. Additional illustrative quotes can be found in Supplementary 1.

### [1] *Prescribing validation: The role of opioids in shaping perceptions of care*

The first main theme explores how opioid prescriptions are perceived by patients, as either markers of recognition or dismissal of their pain experience. This might stem from the fact that patients have knowledge about opioids, that they are provided for acute pain such as post-surgical recovery or injury. Thereby these medications are viewed as effective for pain relief, so when it is offered for chronic pain, it is interpreted by the patient as a sign that the GP recognizes the pain experience. A narrative throughout the interviews was patients experiencing prescriptions as markers of the legitimacy of pain, thus shaping the perception of the GP-patient relationship. The medication was described as tied to the feeling of being understood.

*Interviewer: What were your expectations or wishes for this visit?*

*That he (doctor) would understand me and that I would, once and for all, it would be, how to say, clear, you must have this pain medicine because there is nothing else (P13).*

The above quote implies that the pain is so severe that nothing besides strong pain medication is an option. Patients expect that their GP will understand their pain experiences and validate the extent of it by prescribing medications. Even subtler cues of hesitation to prescribe opioids are interpreted by the patients as mistrust of the pain even when a prescription is ultimately given.

*Nah but ehh, I know what he wants, and he does, what can I say, he does it for my sake, he's a great doctor (long breath) he's taken all the tests I've requested, eh, so I would be a little reassured. Yes, I understand how they think, absolutely educated and so. No, but I mean that if you can manage without it, then you shouldn't have it, it shouldn't be because you are addicted. Eh, sometimes, sometimes I think like this:”Doesn't he believe me, doesn't he think I'm in pain?” That's how we think (pause) that you're classified as an addict (P7).*

Further, there is an interplay between the consultation experience and implicit expectations of pain management. Satisfaction isn't just about receiving opioids, it's about the GP affirming the legitimacy of their pain by being open for requests. Patients assume that GPs would prescribe more pain relief if requested, suggesting that mutual understanding and validation of the pain is tied to opioid prescriptions.

*I thought it was good, simply it felt like she, uhm, she listened to me, we talked a little about history, how it had been and then further ahead. […] The medication we didn't talk about that, and we have talked about that a lot before, because I've had a lot of prescriptions for a long time. So, I don't think it is, if I had said that I wanted more pain relief, I think she would have prescribed more pain medication, because she knows that I, that I know about it. So, I haven't experienced any frustration there (P10).*

A recurring pattern was that not receiving a prescription was often experienced as a form of dismissal or judgment. For some patients the issue is not just the treatment decision itself, but the way the refusal is communicated. Patients expect GPs to be empathetic and supportive; traits they associate with the willingness to prescribe opioids. When opioids are withheld, patients interpret this not only as a clinical judgment. Instead, patients interpret it as a sign of moral judgment from the GP. So, beyond generating unmet expectations and delegitimizing the patient's pain experience, prescription denial can also contribute to a sense of stigmatization.

*What do they suspect? Why do they think I'm totally drugged up? I don't want to be, I take as little medicine as I can, I have my mother in front of me, I don't want that. And to be distrusted instead of what I am used to, doctors who listen to you and take you seriously, they do that in Spain, they are good doctors. I mean, I'm not a person who, but I'm turning 60 this summer and don't think I would want to become a drug addict now. I don't drink coffee, I don't smoke, I don't drink, if I sometimes take some medicine when I'm in pain, is it so dangerous? (P8)*

The process of medication tapering is tied to the patient's perception that their GP does not truly care about their pain condition and the broader consequences it has on their emotional well-being and quality of life. Patients describe the experience as more than just a reduction in medication; it is a withdrawal of support, a dismissal of their suffering.

*Yes, he is, it is, it's all about which medicine I take. Yes, I take, uh, OxyContin. But the only thing he is after is for me to remove the medication. Whatever it costs. Yes, it's hard. It creates great anxiety, and I know what it means to be without the medicine. In my movement and my manner as well, to be able to live a decent life. But it's not interesting to him (P3).*

In summary, the theme explores how patients interpret opioid prescription decisions. An opioid prescription is seen more than just a medical choice; the prescription is symbolic. Receiving prescription can foster a sense of trust, reinforcing the belief that the GP understands and takes their suffering seriously. Conversely, when opioids are denied, patients may feel dismissed or doubted, leading to frustration and mistrust. This theme captures how prescription decisions shape not only treatment outcomes but also the emotional and relational dynamics between patients and GPs.

### [2] *The battle for the steering wheel: The patient's voice in pain management*

The second main theme captures patients' experience of agency in consultations. Divided into two sub-themes it describes how patients expressed both frustration and perceived exclusion from treatment decisions. Patients express a desire to be active participants in their care and how they experience that their voices aren't heard or valued in consultation.

#### Subtheme 2.1 My body, My pain, My choice: Claiming expertise in pain management

This subtheme illustrates how patients navigate tensions between respecting GPs' authority and asserting their own agency in treatment decisions. Resistance can range from direct or active resistance, such as openly disagreeing with the GP's recommendations, to more subtle forms of pushback, like implying dissatisfaction or questioning the GP's decisions. Patients who asserted their personal knowledge about their condition often framed it as an act of self-advocacy. The lived experience is emphasized as a critical component of treatment decisions, particularly when GPs disagree with treatments choices.

When patients oppose opioid tapering, they argue against GPs decisions by asserting their ability to use the medication responsibly. Patients emphasize that their consumption is measured, intentional and based on their body knowledge and dismiss GPs concerns of addiction. All patients recognize the addictive nature of opioids but claim that their life without medication would be a life with grave limitations.

*Then there's always this issue of the painkillers, that yes, it's addictive and everything, but I take such extreme low doses and only use it in the fall and winter when it gets cold, that's when I feel the worst. And then they try to eliminate that too and come up with something else (P6).*

*But it's still like, I think that in order to get a, a, what shall I say, a life where you can move around. Then it can cost whatever. And I can tell you that, the day that they, well, when I have to end it, I will be sitting here at home, I think that is how I will be. If that happens, I won't want to live anymore…. No because I… what am I going to say, well, you get addicted very quickly, maybe already after a couple of weeks. And now, I mean, I've been on this medicine for so long and… well, what can I say? Well, it's clear that it's a disadvantage to be dependent on something, but if I may compare it to, yes, I'm happy to be dependent on this medicine to have a tolerable life (P3).*

Patients understand the challenges GPs are faced with when prescribing opioids but still put forward a rationale why they should have access to larger prescriptions.

*But it's, it's good on a general level, but for me it's hard, I'd rather have, yes, a proper prescription, so that I know that I, I have a prescription if I need to take out medicine, then I don't have to call and pay a hundred crowns (nine euros) for a phone call. And chasing people and getting appointments, so I would have rather seen bigger prescriptions for me, but at the same time I get that it's a bit hard because others who might have it hard have it too (P10).*

Further, the consultations are described as one-sided, where GPs focused on guidelines rather than personalized care.

*I think I've tried so much physiotherapy and I exercise a lot and I kind of have a good handle on my body, so these physiotherapist visits I did this time they kind of gave me nothing. Eh, and I think that's a little frustrating, but I's frustrating that you have to go there (P10).*

A majority of patients describe how they have been living with long-term pain, and how that makes them a specialist on their own body. Some of the patients resist the GPs view of the negative side effects of the medication.

*They don't understand I am actually a specialist of my own body, because you are that when you are this old. And I'm a person who, a normal person who wants to live a good life and I don't want to do drugs, she (GP) said 'I can't put you to sleep'. No and clearly, I don't want to be put to sleep, but you have to understand that I after this long time with so much medicine, I was born like this, I know that I should be able to take my medicine, of course I try to figure it out myself (P8).*

In summary, this sub-theme underscores how patients insert their voice in pain management consultations regarding opioid use and claim that the lived experience is as valid as medical advice from GPs. In essence, patients have expectations based on long term experience of living with pain, which they view as granting them expertise. This is juxtaposed with the GPs role and view of themselves as experts in treating pain, resulting in unmet expectations for the patients.

#### Subtheme 2.2 does my voice matter? – the emotional toll of not being heard

The second subtheme highlights the emotional impact of being dismissed or overlooked. Patients describe not just disagreement with the GP regarding opioids, but a deeper feeling of not being listened to. Patients express how their concerns and worries remain after consultations, leaving them feeling powerless, discouraged, and in some cases, withdrawal from seeking medical help altogether. This experience often reinforces a belief that their voices do not matter in treatment decisions.

One patient illustrates how concerns about opioid use are overlooked. Despite ongoing worries about side effects, the GP fails to address or alleviate these concerns during the consultation:

*P1: Yes, that, it's that I have a pain in my lower back, in my back, which is evident from me saying during the recording that it's like it's, I'm a bit worried about that, therefore, yes I think it's the internal organs that are taking a beating, from the medications, yes, that's why I asked him, 'are the kidneys far down?'*

*Interviewer: Did you believe you got an answer to that?*

*P1: No.*

*Interviewer: okay, mm, so you still have that concern?*

*P1: Yes, I still have it, yes.*

A recurrent pattern was patients describing the struggle to express themselves during the consultation, suggesting a power imbalance or emotional barrier in the interaction. This interaction reflects a lack of meaningful engagement, where the patient's concerns are recognized but not explored, potentially leaving them feeling unheard or invalidated.

*Yes, it (consultation) was good, I didn't get much out of what I wanted or thought, I was tongue-tied, couldn't get out what I wanted to say… I wanted to say that I had a lot more anxiety now and stuff like that,'I think you also have that now' he said and then talked about something else, then he tried to talk about something else (P15).*

Patients emphasize a sense of power imbalance and describe how they as patients don't want to be a burden and consequently don't disclose all their concerns to their GPs. Instead of being frank about their concerns during the consultations, patients actively portray themselves as compliant.

P7: *I had a wish, but I lost it. That I wanted an x-ray on my right hip.*

*Interviewer: Do you have any idea why you forgot it?*

*P7: You don't want to be burden. No but that's kind of how you think, it'll take a while, I'll take it next time or, erm, for now I can walk. But there will (pause) come a day when it (pause) is too much, well then I'll have to go there and be a victim.*

Further, patients suggest expectations are affected by past visits and that the visit leads to a deep sense of frustration and emotional impact, emphasizing a perceived lack of recognition and attentiveness from healthcare providers.

*So those of us who are sick are reluctant to contact a doctor at all, it should be when you feel that you are not doing well at all, then we call, but the thing is that you have gotten so much crap, to say it in plain Swedish, so so then you stop going to the doctor, the worst thing for me when I go to a doctor and he or she questions what I say. You see, about how I'm dealing with my pain, it's absolutely, it's like they're hitting you, because what I say is true. That's how it is. Yes, but I feel it, I'm a person after all, that they barely listen, they can be a bit dismissive, like, yeah, that's how it is (P12).*

In summary this sub-theme captures how concerns are not properly handled during the consultation and that patients either don't get an answer to their question or that they don't even say everything they have in mind because of a feeling one ought not to.

## Discussion

The aim of this study was to explore how patients experience their pain management consultations regarding opioid prescriptions. The findings imply that patients enter these consultations with expectations that go beyond symptom relief. Patients expect acknowledgment of their suffering and expertise in managing their own pain. How GPs respond to these expectations, particularly regarding opioid prescribing decisions, carry symbolic meaning for patients. Opioid prescriptions are not just pharmacological decisions: receiving a prescription from the GP provides a sense of validation of the pain. In contrast, not receiving a prescription is interpreted as an invalidation of the lived experience of pain. Hence, the presence or absence of a prescription shapes patients' emotional responses to their interactions with GPs. The findings highlight the importance of considering expectations as an important aspect of patient-clinician trust and alliance.

### Findings in relation to other studies

Prior qualitative studies have shown that patients find communication with clinicians less than ideal. The interactions are marked with disagreement and lack of mutual trust [51–53]. Patients desire to be listened to [54], and that clinicians show understanding for their complex pain condition and validate their unique pain experience [55]. Henry and Matthias's [56] conceptual model of communication about pain (Figure 1) emphasizes the importance of relational communication for fostering shared decision-making. Further, a robust long-term relationship is a fertile ground for perceiving the clinician as empathetic [53] and is required for shared-decision making to take place [57]. Patients struggle to have their concerns acknowledged, which indicates an absence of relational communication. The consequences for patients are frustration and a diminished sense of agency in their care. The findings therefore reinforce that effective pain management consultations regarding opioid use heavily rely on relational communication to be effective.

The framework of expectations stipulates that patient frustration often emerges from a mismatch between expectations and reality [43]. This study confirms that patients appeared to enter consultations with the idealized expectation that their pain would be acknowledged, validated and that a renewed prescription would follow. When patients were denied opioids without a clear explanation and in a way the patients perceived unsympathetic it violated their ideal expectation of shared decision making. This finding resonates with insights from previous studies suggesting that patients and GPs operate from different expectations regarding long-term opioid therapy: patients consider themselves cautious opioid users and expect their opioid regimen to remain stable unless they experience clear harm. GPs expect patients to consider adverse drug effects, emphasize potential risks and therefore expecting patients to view tapering as favorable and be open to tapering [58,59].

Most of the participants in this study were on a low dose regimen of opioids. However, a few stood out having a higher dose. This variation is expected in this population and reflects what GPs encounter in everyday practice. Hence, the dose variability can be viewed as part of the clinical context. The thematic pattern found in the current study reflected a shared experience of opioid therapy and, independent of dosage, patients described struggling with identity (“I'm not an addict, I'm in pain”), feeling dependent on medication and being afraid of tapering. This indicates a shared underlying experience of living with chronic pain and opioids. Participants with low doses sometimes described “feeling like a drug addict” and high dose participants sometimes described “it's stable and not a big deal”. What thus seems to matter for the participants is how they perceive their dose, not the factual dose.

All in all, the results of this study indicate that patients' views are quite different from what is the GP's perspective, who may consider the ongoing opioid crisis in the United States [60] and struggle to relate to complex prescribing directives [61]. Important concerns for GPs are to adhere to guidelines, and to prevent negative side effects and addiction, while also maintaining a good patient-doctor relationship [61]. Requests for opioids, unrealistic expectations, and refusal to try recommended treatments have all been reported by clinicians as factors contributing to difficult encounters with chronic pain patients [62]. Misaligned expectations between patients and GPs can be key drivers of the communication and decision-making challenges resulting in patients feeling invalidated and unheard. These dynamics place communication at the core of care: empathy must be balanced with boundary-setting and risks clearly explained, especially if the dose regime is viewed differently from the patients' lens.

In line with previous research the patients in this study described their lived experience of pain as granting them a form of expertise in pain management, positioning them as credible voices in discussions about their care [29]. This sense of expertise was particularly evident when patients felt their insights were dismissed or overshadowed by GPs perspectives, thus reinforcing a perceived divide between their knowledge based on experience and the medical expertise and authority of GPs. A key feature of this pattern is that patients seek validation for their pain experience. This pattern has been noted in previous studies capturing chronic pain patients' experiences: patients seek explanations for the symptoms, a diagnosis and they tend to view their pain through a biomedical lens [63,64]. Since recent research suggests that a proportion of medically unexplained pain symptoms can be understood as nociplastic pain [17], a lot can be gained from facilitating patients' comprehension of this complex phenomenon and thereby validating the pain condition.

In summary, the current study underscores the importance of empathetic communication in pain management regarding opioid use. However, the result also shows that patient-clinician relations are expectation-driven, underscoring the importance of clear communication regarding ways to manage opioid renewals for chronic pain patients as well as a need to ensure that pain consultations are informed by up-to-date guidelines and shared frameworks for the understanding of chronic pain.

### Strengths and limitations

The strength and aim of this study were to focus on patient narratives. The all-encompassing perspective was a constructivist approach with the aim of getting as close as possible to the patients' personal narratives regarding their experiences. The themes constructed display a reflexive process of the author engaging with the ways patients themselves construct meaning in their medical encounters. As such, these findings may be applicable to comparable clinical contexts, but not necessarily those that differ socio-culturally or economically.

This study also has limitations that need to be addressed. First, patients who chose to participate may differ from those who declined, for instance in terms of willingness to reflect on their experiences and/or how comfortable and/or motivated they were to share their views. The overall study included that the consultations would be recorded, and there were patients who declined to participate due to not wanting to be captured on film. Additionally, there is a risk that both the patients and the GPs were constrained by the fact that the consultation was recorded. However, existing research on video recorded physician–patient interactions has not demonstrated any notable behavioral changes attributable to Hawthorne effects [65,66]. Further, although the perspectives captured may not encompass the full spectrum of patient experiences, the rich and detailed accounts provide insight into how patients understand and engage in the consultations.

Second, the interview was conducted over phone by a GP, which can be seen as a limitation. There is a risk the GP interviewer was perceived as an authority, creating a power imbalance and leading to the patients restraining themselves in the interviews. However, the result indicates the patients opened up and were frank about their experience. Drawing on Foucault's and Bourdieu's theories, such imbalances can tentatively be interpreted as fluidly co-constructed power dynamics, here premised on professional status differences, perpetuated in the interview context [67]. As to the fact that the interviews were held over phone this must be considered a limitation. Facial expressions and other non-verbal expressions are lost, and research shows that in-person interviews are preferred [68]; however, due to practical issues, telephone was the only option. Additionally, due to the same practical constraints, member reflection was not included in this study design. This could have been a useful adjunct, as it might have offered some valuable perspective and compiled extra data illuminating different viewpoints between the participants and the written recount [69]. However, the final report and the findings will be shared with the patients.

Third, even if the interviews were conducted closely in time after the consultation, they do not fully capture the experience in the moment. Immediacy of emotions and experiences may fade or be reframed, hence impacting how patients reconstruct their expectations and interactions. Furthermore, since expectations were measured retrospectively there were no ways to compare what the patient anticipated before the consultation. A pre- and post-interview design would provide a clearer picture of the patients' expectations.

## Implications for research and practice

A main finding from the current study was the insight that patients view prescription withdrawal as an invalidation of the pain. This is especially the case when decision was made without clear and empathetic reasoning. Patients' expectations can be better met by finding ways to validate patients' pain even when opioids are not prescribed, such as through empathy, clear communication, and offering alternative options. Hence, a robust GP-patient relationship is of great value and earlier research suggests that targeted training in communication and diagnostic reasoning could help reduce unnecessary opioid prescribing [70]. Actively asking patients about their expectations regarding opioids, ascertain the patients understanding of the opioid usage and explaining reasons why the GP do not share the same view is important, knowing that patients do not consistently share the same understanding regarding the negative effects of opioids as mentioned above. In conjugation with facilitating patients understanding of pain and thoroughly explaining nociplastic pain could be one way to validate the patients' pain experience.

In the Swedish healthcare context there is an ongoing attempt to provide the means for a regular and longstanding relationship between GP and patient [71]. One might argue there could be other representatives in the healthcare system that could fill the role as stable center points and form long lasting and strong relationships with patients. Insight from research on depression care in primary care could contribute with knowledge. To be able to offer steady and accessible healthcare, efforts have been made to incorporate a *care manager*, often nurses who act as liaisons between patients and primary care GPs. This strategy has been successful and resulted in better outcomes for the patient [72].

The current study has focused on how patients experience their pain management consultations regarding opioid use. Nevertheless, patient narratives are not constructed in isolation, they are shaped by broader societal and medical discourses surrounding chronic pain, opioid use, and healthcare interactions. For example, the stigma surrounding opioid prescriptions, societal skepticism toward chronic pain sufferers, often reinforced by media, might influence how patients justify, defend, or narrate their pain experiences and their medication needs. Further research focusing on how these discourses shape patients' narratives could contribute to the understanding of how patients experience communication and expectations of pain management consultations.

## Supplementary Material

Supplementary 2 interview guide.docx

Reflexivity Statement.docx

Supplementary 1 Additional Illustrative Quotes by Theme.docx

## References

[1] Breivik H, Collett B, Ventafridda V, et al. Survey of chronic pain in Europe: prevalence, impact on daily life, and treatment. Eur J Pain. 2006;10(4):287–333. doi: 10.1016/j.ejpain.2005.06.009. 16095934

[2] Dahlhamer J, Lucas J, Zelaya C, et al. Prevalence of chronic pain and high-impact chronic pain among adults—United States, 2016. MMWR Morb Mortal Wkly Rep. 2018;67(36):1001–1006. doi: 10.15585/mmwr.mm6736a2. 30212442 PMC6146950

[3] Rometsch C, Martin A, Junne F, et al. Chronic pain in European adult populations: a systematic review of prevalence and associated clinical features. Pain. 2025;166(4):719–731. doi: 10.1097/j.pain.0000000000003406. 40101218 PMC11921450

[4] Deyo RA, Mirza SK, Martin BI. Back pain prevalence and visit rates: estimates from US national surveys, 2002. Spine (Phila Pa 1976). 2006;31(23):2724–2727. doi: 10.1097/01.brs.0000244618.06877.cd. 17077742

[5] Hasselström J, Liu-Palmgren J, Rasjö-Wrååk G. Prevalence of pain in general practice. Eur J Pain. 2002;6(5):375–385. doi: 10.1016/s1090-3801(02)00025-3. 12160512

[6] Shetty S, Scuffell J, Aitken D, et al. Chronic pain-prevalence, demographic inequalities and healthcare utilisation: a primary care database analysis. BJGP Open. 2025;9(3):BJGPO.2024.0147. doi: 10.3399/BJGPO.2024.0147. 40015745 PMC12728837

[7] Hafezparast N, Bragan Turner E, Dunbar-Rees R, et al. Identifying populations with chronic pain in primary care: developing an algorithm and logic rules applied to coded primary care diagnostic and medication data. BMC Prim Care. 2023;24(1):184. doi: 10.1186/s12875-023-02134-1. 37691103 PMC10494405

[8] Fayaz A, Ayis S, Panesar SS, et al. Assessing the relationship between chronic pain and cardiovasculardisease: a systematic review and meta-analysis. Scand J Pain. 2016;13(1):76–90. doi: 10.1016/j.sjpain.2016.06.005. 28850537

[9] Reynolds CA, Minic Z. Chronic pain-associated cardiovascular disease: the role of sympathetic nerve activity. Int J Mol Sci. 2023;24(6):5378. doi: 10.3390/ijms24065378. 36982464 PMC10049654

[10] Jennifer S, Brady BR, Ibrahim MM, et al. Co-occurrence of chronic pain and anxiety/depression symptoms in US adults: prevalence, functional impacts, and opportunities. Pain. 2024;165(3):666–673. 37733475 10.1097/j.pain.0000000000003056 PMC10859853

[11] Aaron RV, Ravyts SG, Carnahan ND, et al. Prevalence of depression and anxiety among adults with chronic pain: a systematic review and Meta-Analysis. JAMA Netw Open. 2025;8(3):e250268-e. doi: 10.1001/jamanetworkopen.2025.0268. 40053352 PMC11889470

[12] Beyera GK, O'Brien J, Campbell S. Health-care utilisation for low back pain: a systematic review and meta-analysis of population-based observational studies. Rheumatol Int. 2019;39(10):1663–1679. doi: 10.1007/s00296-019-04430-5. 31463608

[13] Mose S, Kent P, Smith A, et al. Trajectories of musculoskeletal healthcare utilization of people with chronic musculoskeletal pain–a population-based cohort study. Clin Epidemiol. 2021;13:825–843. doi: 10.2147/CLEP.S323903. 34557040 PMC8455515

[14] Emilson C, Åsenlöf P, Demmelmaier I, et al. Association between health care utilization and musculoskeletal pain. A 21-year follow-up of a population cohort. Scand J Pain. 2020;20(3):533–543. doi: 10.1515/sjpain-2019-0143. 32755105

[15] Meroni R, Piscitelli D, Ravasio C, et al. Evidence for managing chronic low back pain in primary care: a review of recommendations from high-quality clinical practice guidelines. Disabil Rehabil. 2021;43(7):1029–1043. doi: 10.1080/09638288.2019.1645888. 31368371

[16] Kosek E, Clauw D, Nijs J, et al. Chronic nociplastic pain affecting the musculoskeletal system: clinical criteria and grading system. Pain. 2021;162(11):2629–2634. doi: 10.1097/j.pain.0000000000002324. 33974577

[17] Fitzcharles M-A, Cohen SP, Clauw DJ, et al. Nociplastic pain: towards an understanding of prevalent pain conditions. Lancet. 2021;397(10289):2098–2110. doi: 10.1016/S0140-6736(21)00392-5. 34062144

[18] Nijs J, Lahousse A, Kapreli E, et al. Nociplastic pain criteria or recognition of central sensitization? Pain phenotyping in the past, present and future. J Clin Med. 2021;10(15):3203. doi: 10.3390/jcm10153203. 34361986 PMC8347369

[19] Läkemedelsverket. Läkemedelsbehandling av långvarig smärta hos barn och vuxna behandlingsrekommendation; 2017.

[20] Häuser W, Morlion B, Vowles KE, et al. European* clinical practice recommendations on opioids for chronic noncancer pain–Part 1: role of opioids in the management of chronic noncancer pain. Eur J Pain. 2021;25(5):949–968. doi: 10.1002/ejp.1736. 33655607 PMC8248186

[21] Cheung CW, Qiu Q, Choi S-W, et al. Chronic opioid therapy for chronic non-cancer pain: a review and comparison of treatment guidelines. Pain Phys. 2014;5;17(5;9):401–414. doi: 10.36076/ppj.2014/17/401. 25247898

[22] Häuser W, Buchser E, Finn DP, et al. Is Europe also facing an opioid crisis?—A survey of European Pain Federation chapters. Eur J Pain. 2021;25(8):1760–1769. doi: 10.1002/ejp.1786. 33960569

[23] Jayawardana S, Forman R, Johnston-Webber C, et al. Global consumption of prescription opioid analgesics between 2009-2019: a country-level observational study. EClinicalMedicine. 2021;42:101198. doi: 10.1016/j.eclinm.2021.101198. 34820610 PMC8599097

[24] Muller AE, Clausen T, Sjøgren P, et al. Prescribed opioid analgesic use developments in three Nordic countries, 2006–2017. Scand J Pain. 2019;19(2):345–353. doi: 10.1515/sjpain-2018-0307. 30677000

[25] Ashaye T, Hounsome N, Carnes D, et al. Opioid prescribing for chronic musculoskeletal pain in UK primary care: results from a cohort analysis of the COPERS trial. BMJ Open. 2018;8(6):e019491. doi: 10.1136/bmjopen-2017-019491. PMC6009475 29880563

[26] Busse JW, Wang L, Kamaleldin M, et al. Opioids for chronic noncancer pain: a systematic review and meta-analysis. Jama. 2018;320(23):2448–2460. doi: 10.1001/jama.2018.18472. 30561481 PMC6583638

[27] Chou R, Turner JA, Devine EB, et al. The effectiveness and risks of long-term opioid therapy for chronic pain: a systematic review for a National Institutes of Health Pathways to Prevention Workshop. Ann Intern Med. 2015;162(4):276–286. doi: 10.7326/M14-2559. 25581257

[28] Eriksen J, Sjøgren P, Bruera E, et al. Critical issues on opioids in chronic non-cancer pain:: an epidemiological study. Pain. 2006;125(1-2):172–179. doi: 10.1016/j.pain.2006.06.009. 16842922

[29] Esquibel AY, Borkan J. Doctors and patients in pain: conflict and collaboration in opioid prescription in primary care. PAIN^®^. 2014;155(12):2575–2582. doi: 10.1016/j.pain.2014.09.018. 25261714

[30] Kennedy LC, Binswanger IA, Mueller SR, et al. “Those conversations in my experience don't go well”: a qualitative study of primary care provider experiences tapering long-term opioid medications. Pain Med. 2018;19(11):2201–2211. doi: 10.1093/pm/pnx276. 29126138 PMC6454789

[31] Thomas KH, Dalili MN, Cheng H-Y, et al. Prevalence of problematic pharmaceutical opioid use in patients with chronic non‐cancer pain: a systematic review and meta‐analysis. Addiction. 2024;119(11):1904–1922. doi: 10.1111/add.16616. 39111346

[32] Benyamin R, Trescot AM, Datta S, et al. Opioid complications and side effects. Pain Physician. 2008;11(2 Suppl):S105–S120. doi: 10.36076/ppj.2008/11/S105. 18443635

[33] De Sola H, Maquibar A, Failde I, et al. Living with opioids: a qualitative study with patients with chronic low back pain. Health Expect. 2020;23(5):1118–1128. doi: 10.1111/hex.13089. 32558064 PMC7696128

[34] Ljungvall H, Rhodin A, Wagner S, et al. “My life is under control with these medications”: an interpretative phenomenological analysis of managing chronic pain with opioids. BMC Musculoskelet Disord. 2020;21(1):61. doi: 10.1186/s12891-020-3055-5. 32005212 PMC6995209

[35] Henry SG, Matthias MS. Patient-clinician communication about pain: a conceptual model and narrative review. Pain Med. 2018;19(11):2154–2165. doi: 10.1093/pm/pny003. 29401356 PMC6454797

[36] McDonnell E, Harmon D. Chronic pain patients' perceptions of prescription opioids: a systematic review. SN Compr Clin Med. 2020;2(12):2816–2824. doi: 10.1007/s42399-020-00599-0.

[37] Schmitz J, Müller M, Stork J, et al. Positive treatment expectancies reduce clinical pain and perceived limitations in movement ability despite increased experimental pain: a randomized controlled trial on sham opioid infusion in patients with chronic back pain. Psychother Psychosom. 2019;88(4):203–214. doi: 10.1159/000501385. 31302644

[38] Montag LT, Bisson EJ, Duggan S, et al. Patient expectations and therapeutic alliance affect pain reduction following lidocaine infusion in an interdisciplinary chronic pain clinic. J Pain. 2024;25(6):104443. doi: 10.1016/j.jpain.2023.11.026. 38056545

[39] Peerdeman KJ, van Laarhoven AIM, Keij SM, et al. Relieving patients' pain with expectation interventions: a meta-analysis. Pain. 2016;157(6):1179–1191. doi: 10.1097/j.pain.0000000000000540. 26945235

[40] Cormier S, Lavigne GL, Choinière M, et al. Expectations predict chronic pain treatment outcomes. Pain. 2016;157(2):329–338. doi: 10.1097/j.pain.0000000000000379. 26447703

[41] Matthias MS, Donaldson MT, Jensen AC, et al. I was a little surprised”: qualitative insights from patients enrolled in a 12-month trial comparing opioids with nonopioid medications for chronic musculoskeletal pain. J Pain. 2018;19(9):1082–1090. doi: 10.1016/j.jpain.2018.04.008. 29715520 PMC6349028

[42] Matthias MS, Johnson NL, Shields CG, et al. “I'm not gonna pull the rug out from under you”: Patient-provider communication about opioid tapering. J Pain. 2017;18(11):1365–1373. doi: 10.1016/j.jpain.2017.06.008. 28690000 PMC6219456

[43] Geurts JW, Willems PC, Lockwood C, et al. Patient expectations for management of chronic non‐cancer pain: a systematic review. Health Expect. 2017;20(6):1201–1217. doi: 10.1111/hex.12527. 28009082 PMC5689237

[44] Thompson AG, Suñol R. Expectations as determinants of patient satisfaction: concepts, theory and evidence. Int J Qual Health Care. 1995;7(2):127–141. doi: 10.1093/intqhc/7.2.127. 7655809

[45] Kravitz RL. Patients' expectations for medical care: an expanded formulation based on review of the literature. Med Care Res Rev. 1996;53(1):3–27. doi: 10.1177/107755879605300101. 10156434

[46] Wiering B, de Boer D, Krol M, et al. Entertaining accurate treatment expectations while suffering from chronic pain: an exploration of treatment expectations and the relationship with patient-provider communication. BMC Health Serv Res. 2018;18(1):706. doi: 10.1186/s12913-018-3497-8. 30200955 PMC6131883

[47] Kallio H, Pietilä AM, Johnson M, et al. Systematic methodological review: developing a framework for a qualitative semi‐structured interview guide. J Adv Nurs. 2016;72(12):2954–2965. doi: 10.1111/jan.13031. 27221824

[48] Braun V, Clarke V. Using thematic analysis in psychology. Qual Res Psychol. 2006;3(2):77–101. doi: 10.1191/1478088706qp063oa.

[49] Braun V, Clarke V. Conceptual and design thinking for thematic analysis. Qual Psychol. 2022;9(1):3–26. doi: 10.1037/qup0000196.

[50] Braun V, Clarke V. To saturate or not to saturate? Questioning data saturation as a useful concept for thematic analysis and sample-size rationales. Qual Res Sport, Exer Health. 2021;13(2):201–216. doi: 10.1080/2159676X.2019.1704846.

[51] Upshur CC, Bacigalupe G, Luckmann R. “They don't want anything to do with you”: Patient views of primary care management of chronic pain. Pain Med. 2010;11(12):1791–1798. doi: 10.1111/j.1526-4637.2010.00960.x. 21029353

[52] Buchman DZ, Ho A, Illes J. You present like a drug addict: patient and clinician perspectives on trust and trustworthiness in chronic pain management. Pain Med. 2016;17(8):1394–1406. doi: 10.1093/pm/pnv083. 26759389 PMC4975016

[53] Dassieu L, Heino A, Develay É, et al. “They think you're trying to get the drug”: Qualitative investigation of chronic pain patients' health care experiences during the opioid overdose epidemic in Canada. Can J Pain. 2021;5(1):66–80. doi: 10.1080/24740527.2021.1881886. 34189391 PMC8210863

[54] Haverfield MC, Giannitrapani K, Timko C, et al. Patient-centered pain management communication from the patient perspective. J Gen Intern Med. 2018;33(8):1374–1380. doi: 10.1007/s11606-018-4490-y. 29845465 PMC6082206

[55] Evers S, Hsu C, Sherman KJ, et al. Patient perspectives on communication with primary care physicians about chronic low back pain. Perm J. 2017;21(4):16–177. doi: 10.7812/TPP/16-177. PMC5638625 29035178

[56] Henry SG, Bell RA, Fenton JJ, et al. Communication about chronic pain and opioids in primary care: impact on patient and physician visit experience. Pain. 2018;159(2):371–379. doi: 10.1097/j.pain.0000000000001098. 29112009 PMC5934342

[57] Matthias MS, Henry SG. Reducing frustration and improving management of chronic pain in primary care: is shared decision-making sufficient? J Gen Intern Med. 2022;37(1):227–228. doi: 10.1007/s11606-021-06967-3. 34173195 PMC8739407

[58] Anderson TS, Wang BX, Lindenberg JH, et al. Older adult and primary care practitioner perspectives on using, prescribing, and deprescribing opioids for chronic pain. JAMA Netw Open. 2024;7(3):e241342-e. doi: 10.1001/jamanetworkopen.2024.1342. 38446478 PMC10918495

[59] Nevedal AL, Timko C, Lor MC, et al. Patient and provider perspectives on benefits and harms of continuing, tapering, and discontinuing long-term opioid therapy. J Gen Intern Med. 2023;38(8):1802–1811. doi: 10.1007/s11606-022-07880-z. 36376623 PMC9663196

[60] Dowell D, Haegerich TM, Chou R. CDC guideline for prescribing opioids for chronic pain—United States, 2016. Jama. 2016;315(15):1624–1645. doi: 10.1001/jama.2016.1464. 26977696 PMC6390846

[61] Punwasi R, De Kleijn L, Rijkels-Otters J, et al. General practitioners' attitudes towards opioids for non-cancer pain: a qualitative systematic review. BMJ Open. 2022;12(2):e054945. doi: 10.1136/bmjopen-2021-054945. PMC8808445 35105588

[62] Cohen SP, Liu WL, Doshi TL, Wang EJ, van Gelderen E, Mawalkar R, et al. editors. Difficult encounters in chronic pain patients: a cohort study. Mayo Clinic Proceedings. 2025;100(1):30–41. doi: 10.1016/j.mayocp.2024.08.010. 39641718

[63] Keen S, Lomeli-Rodriguez M, Williams A. Exploring how people with chronic pain understand their pain: a qualitative study. Scand J Pain. 2021;21(4):743–753. doi: 10.1515/sjpain-2021-0060. 34331751

[64] Toye F, Seers K, Hannink E, et al. A mega-ethnography of eleven qualitative evidence syntheses exploring the experience of living with chronic non-malignant pain. BMC Med Res Methodol. 2017;17(1):116. doi: 10.1186/s12874-017-0392-7. 28764666 PMC5540410

[65] Henry SG, Jerant A, Iosif A-M, et al. Analysis of threats to research validity introduced by audio recording clinic visits: selection bias, Hawthorne effect, both, or neither? Patient Educ Couns. 2015;98(7):849–856. doi: 10.1016/j.pec.2015.03.006. 25837372 PMC4430356

[66] Themessl-Huber M, Humphris G, Dowell J, et al. Audio-visual recording of patient–GP consultations for research purposes: a literature review on recruiting rates and strategies. Patient Educ Couns. 2008;71(2):157–168. doi: 10.1016/j.pec.2008.01.015. 18356003

[67] Aléx L, Hammarström A. Shift in power during an interview situation: methodological reflections inspired by Foucault and Bourdieu. Nurs Inq. 2008;15(2):169–176. doi: 10.1111/j.1440-1800.2008.00398.x. 18476859

[68] Johnson DR, Scheitle CP, Ecklund EH. Beyond the in-person interview? How interview quality varies across in-person, telephone, and Skype interviews. Social Science Computer Review. 2021;39(6):1142–1158. doi: 10.1177/0894439319893612.

[69] Braun V, Clarke V. Toward good practice in thematic analysis: Avoiding common problems and be (com) ing a knowing researcher. Int J Transgend Health. 2023;24(1):1–6. doi: 10.1080/26895269.2022.2129597. 36713144 PMC9879167

[70] Tamblyn R, Girard N, Boulet J, et al. Association of clinical competence, specialty and physician country of origin with opioid prescribing for chronic pain: a cohort study. BMJ Qual Saf. 2022;31(5):340–352. doi: 10.1136/bmjqs-2021-013503. PMC9046738 34725228

[71] Myndigheten för vård- och omsorgsanalys. Fast kontakt i primärvården. Patienters uppfattning om tillgången till fast läkarkontakt och fast vårdkontakt i primärvården. Stockholm: myndigheten för vård- och omsorgsanalys; 2021. Report No.: PM 2021:1.

[72] Björkelund C, Svenningsson I, Hange D, et al. Clinical effectiveness of care managers in collaborative care for patients with depression in Swedish primary health care: a pragmatic cluster randomized controlled trial. BMC Fam Pract. 2018;19(1):28. doi: 10.1186/s12875-018-0711-z. 29426288 PMC5807835

