# Supplementary material for: Expectations and communication in opioid pain management: a qualitative study of patients' experience
Source: Scand J Prim Health Care. 2026 Jan 23;44(1):2616517. doi: 10.1080/02813432.2026.2616517 (PMC12833907; doi:10.1080/02813432.2026.2616517)
Supplement: Supplementary 2 interview guide.docx [file IPRI_A_2616517_SM2512.docx]

Supplementary 2. Interview guide

| Topic Area | General Interview Questions | Sub- and Follow-up Questions |
| --- | --- | --- |
| 1. The patient’s overall experience of the consultation | You met with your doctor on _______day, how did you experience the visit? | Can you elaborate? What is it like to talk about your symptoms with your doctor? What is it like to talk about your treatment with your doctor? Was there anything you found difficult to talk about? Why? How could it have been easier? Was there anything you talked about that you thought about afterward? |
| 2. The patient's pain experience and understanding of pain | Can you describe the nature of your pain? | Can you tell me about your problem?  What do you think is causing the pain?  If referring to what the doctor said: What do you think about it?  Can you elaborate?  What has the pain done to you and your life?  Have you talked to your doctor about it? How did it go / why not?  How does the pain affect how you feel?  How does the pain affect what you can do?  What is important for the doctor to know about you?  What do you think about when you think about the future?  Have you talked to your doctor about it? How did it go / why not? |
| 3. The patient's expectations and goals regarding the visit. | What were your expectations/wishes for the visit? | Before the visit, was there anything you were thinking about or was worried about that you wanted to bring up?  What?  Did the conversation turn out the way you had hoped?  Did you have any hopes or wishes for changes to your treatment, if so, what?  To what extent did you feel that your doctor agreed to your wishes? 1-10. Opens for follow-up question to capture dissatisfaction: Why 3 and not 5, every 3 and not 0?  Was there anything in particular in the meeting that you have in mind when you say this? |
| 4. The patient's expectations and goals of pharmacological pain treatment. | How do you feel about the medications you’re on? | What is the reason you are receiving them, what is the purpose of the treatment?  What effect do they have?  What are the advantages of ______?  What are the disadvantages of ______?  How do you think medication can affect your situation?  What can you do that you could not do without ______?  Besides medication, what helps you manage your pain? Can you develop? |
| 5. The ppatient’s concerns and fears, specifically around opioids and addiction | Is there anything about your medications that you are worried about, if so, what?  Do you have any concerns or fears about ________________, what is it about? | Have you talked to your doctor about it?  Do you feel that the doctor understands it? Can you elaborate?  Have you talked to your doctor about it?  How did it turn out?  How would you like it to be?  What do you think about the risk of developing an addiction to __________? |
| 6. Decision-making and relational communication | How is the planning of your care organized? | How are decisions regarding your treatment handled?  How does it affect you?  How would you like the planning of your care to be carried out?  What are some things you think, but don't tell your doctor? |
| 7. Other reflections | How do you feel you were affected by the fact that your doctor's visit was recorded?  Is there anything else you would like to say before we finish? |  |
