# Supplementary material for: Expectations and communication in opioid pain management: a qualitative study of patients' experience
Source: Scand J Prim Health Care. 2026 Jan 23;44(1):2616517. doi: 10.1080/02813432.2026.2616517 (PMC12833907; doi:10.1080/02813432.2026.2616517)
Supplement: Reflexivity Statement.docx [file IPRI_A_2616517_SM2511.docx]

**Reflexivity Statement**

Jennifer Amin, primary analysist.

**Personal, Professional Background and Positionality**

I am a clinical psychologist and PhD student living in a mid-sized city in Sweden. I entered research without any clinical experience regarding chronic pain patients. However, I received my education at Örebro University where research on chronic pain has a prominent role in the faculty. As such, I have an academic familiarity with the topic, and with the biopsychosocial model, which has since shaped my understanding of pain as a multidimensional phenomenon.

My understanding of the consultation context was shaped by personal relationships rather than firsthand professional practice. I am married to a physician who encounters patients with pain in his clinical work. In addition, my mother has been diagnosed with fibromyalgia, and my father suffers from chronic lower back pain.

Many of the participants’ accounts resonated with situations I had encountered in conversations with my mother and father. I noticed a tendency to empathise with patients’ perspectives. In some ways I was deeply engaged in the patients’ narratives and their struggles. However, I also had the perspective of the physicians through discussions with my husband. This perspective normalised certain aspects of the consultation experience that participants described as frustrating or dismissive. I approached the analysis with an ongoing awareness that these two perspectives could colour my reading of the data. Reflexivity was central to the process, and I remained conscious throughout that these two viewpoints might inform, or even bias, my interpretations.

These dual perspectives gave me an acute awareness of positionality. I try to handle this issue in many ways. Firstly, by grounding the study in epistemological constructivism, that emphasizes that knowledge is not passively received but actively constructed. The dual perspectives also helped me hold contradictory and competing information and helped me to analyse ambiguous and conflicting data.

Recognising this I kept a reflexive journal throughout the process to document my reactions, assumptions, and evolving interpretations. Regular conversations with my supervisors and co-authors played a key role in challenging my thinking and broadening my analytical lens. These reflexive practices helped ensure that the themes developed were not simply a reflection of my own worldview, but a careful and critical engagement with the data itself.
