# Supplementary material for: Expectations and communication in opioid pain management: a qualitative study of patients' experience
Source: Scand J Prim Health Care. 2026 Jan 23;44(1):2616517. doi: 10.1080/02813432.2026.2616517 (PMC12833907; doi:10.1080/02813432.2026.2616517)
Supplement: Supplementary 1 Additional Illustrative Quotes by Theme.docx [file IPRI_A_2616517_SM2510.docx]

**Supplementary 1**

*Additional Illustrative Quotes by Theme*

| **Theme**  **Prescribing Validation: The Role of Opioids in Shaping Perceptions of Care** | **Quote**  ”It (consultation) was perfectly fine, that’s pretty much how it usually is if it’s a reasonably good physician. It was no fuss, but but this thing with the meds that we always talk about like, sometimes I don’t keep up but it was good, 9 points out of a possible 10…. I’m a very straightforward person and I think it’s very important to address things that happened, things that didn’t work, for example this when I had, when I was worried that I wouldn’t get medicine for the weekend, I made an appointment with the nurse, and she was going to remind him (physician)… He didn’t really get the information about it in good time if I hadn’t received my medicine and then it would have been hospital next, because this is a big concern, this with the methadone... Takes a lot of my strength when it doesn’t work because it's important medications.” P12  ”[...]it’s probably a way for him to push me down, to sit on me and show that here I’m the one who decides and that was a bit of what I felt the whole visit. And then during the visit he said several times that your pain sits here and then he picks up his head, scratching it like a monkey, or what to say and I didn’t think about that during, but I thought about it when I got home that the way he did it is a bit demeaning, that he doesn’t listen, his way of expressing himself, I kept getting this feeling that, what can I say, here I’m in charge, diminishing me, but he’s always had this input that, that I don’t need the medicine because I’m not in pain. Eh he, so when you say something that is difficult, he keeps relating it to something else or starts talking about something completely different, so it’s a good way to diminish the patient, trick the patient, that’s what he does, or tries to trick you into you’re not in pain, there’s no pain, there’s nothing you can be in pain about, it’s just in your head and everyone knows that it doesn’t work that way.” P13  ”They think remove everything altogether, to see how I react. Remove everything together. All morphine. Other than that I have nothing. yes it has been hard.” P15 |
| --- | --- |
|  | |
| **The Battle for the Steering Wheel: The Patients voice in Pain Management**  **Sub-theme 2.1 My body, My pain, My Choice: Claiming Expertise in Pain Management** | ”Then you should have withdrawal symptoms and there is no one who can see something like that with me, the disadvantages are becoming addicted, eh, and that’s nothing I’ve ever experienced, during certain periods I haven’t taken anything, I, I think it was the summer of 2018 or when it was so terribly hot then I didn’t take much at all, it was about a week I didn’t take a pill, and, but they didn’t believe that at the health center.” P13  ”And I feel that I have so much pain, because it is enough just to look a little to the right and it snaps in my back and shoulder. Yes, and what I noticed when I carried the girl here last Sunday that it only gets worse. And yesterday I actually had a hell of a day, but then these pills still help. These morphine pills in this case, you get addicted. But then again, I haven’t felt anything of that in my head.” P5  ”Well, now we should check something else here because, I, I, have taken these damn pills and there isn’t much to choose from in my situation, you know. No, and, and, and, in order for me to be able to have a tolerable life, I have taken them to make it work, so to speak. Yes, and, because I want to be active, if I can’t be active then, I then told her, then you pull the rug out from under me. Then, then my joy in life is totally to hell. Yes. And, and, it’s so that I can have it as I always had it, right?” P4 |
| **Sub-theme 2.2 Does my voice matter? -- The Emotional Toll of Not Being Heard** | ”No, it was also the expectation that they might have some kind of way of seeing or listening, to take what I say seriously, listen to,yes, listen to it without questioning everything I say. I don’t have a goal in life to sit drugged up in a corner, that’s not what I want with my life. What I want them to listen to when I say ‘I don't feel well’.” P8  ”Well, the only thing on his mind is to stop and prescribe the medicine for me. Yes, he (the physician) is not interested in knowing anything about me. “P3  ”Yes, he is a big idiot who does not listen to me and does not take part of what is written in the records, that both the physiotherapy and the orthopedic surgeon, what they have said. He doesn’t want to listen to that.” P13 |

Note. Quotes are anonymized and identified by participant ID and role. Full theme descriptions are presented in the main manuscript.
